# Drought response of *Mucuna pruriens* (L.) DC. inoculated with ACC deaminase and IAA producing rhizobacteria

**DOI:** 10.1371/journal.pone.0191218

**Published:** 2018-02-15

**Authors:** Aansa Rukya Saleem, Cecilia Brunetti, Azeem Khalid, Gianni Della Rocca, Aida Raio, Giovanni Emiliani, Anna De Carlo, Tariq Mahmood, Mauro Centritto

**Affiliations:** 1 Department of Earth and Environmental Sciences, Bahria University Islamabad Campus, Islamabad, Pakistan; 2 Tree and Timber Institute, National Research Council of Italy, Via Madonna del Piano, Sesto Fiorentino, Firenze, Italy; 3 Dipartimento di Scienze delle Produzioni Agroalimentari e dell'Ambiente, University of Florence, Viale delle Idee Sesto Fiorentino, Firenze, Italy; 4 Department of Environmental Science, Pir Mehr Ali Shah Arid and Agriculture University, Rawalpindi, Pakistan; 5 Institute for Sustainable Plant Protection, National Research Council of Italy, Via Madonna del Piano, Sesto Fiorentino, Firenze, Italy; 6 Islamic Educational, Scientific and Cultural Organization, Rabat, Morocco; Texas Tech University, UNITED STATES

## Abstract

Drought is one of the major constraints limiting agricultural production worldwide and is expected to increase in the future. Limited water availability causes significant effects to plant growth and physiology. Plants have evolved different traits to mitigate the stress imposed by drought. The presence of plant growth-promoting rhizobacteria (PGPR) could play an important role in improving plant performances and productivity under drought. These beneficial microorganisms colonize the rhizosphere of plants and increase drought tolerance by lowering ethylene formation. In the present study, we demonstrate the potential to improve the growth of velvet bean under water deficit conditions of two different strains of PGPR with ACCd (1-Aminocyclopropane-1-Carboxylate deaminase) activity isolated from rainfed farming system. We compared uninoculated and inoculated plants with PGPR to assess: a) photosynthetic performance and biomass; b) ACC content and ethylene emission from leaves and roots; c) leaf isoprene emission. Our results provided evidence that under drought conditions inoculation with PGPR containing the ACCd enzyme could improve plant growth compared to untreated plants. Ethylene emission from roots and leaves of inoculated velvet bean plants was significantly lower than uninoculated plants. Moreover, isoprene emission increased with drought stress progression and was higher in inoculated plants compared to uninoculated counterparts. These findings clearly illustrate that selected PGPR strains isolated from rainfed areas could be highly effective in promoting plant growth under drought conditions by decreasing ACC and ethylene levels in plants.

## Introduction

Drought is the main cause limiting crop production over large areas of Earth. The shifts in rainfall patterns in drought-stricken areas are predicted to increase in the coming decades owing to climate change, with negative effects on plant productivity and extensive losses to agricultural production [1]. Plants respond to water stress through a variety of physiological and morphological changes, which include effects on carbon metabolism, water relations and hormone production that in turn regulate below- and above-ground plant growth [2–4]. Among these responses, increased ethylene production is one of the most common.

Plant growth-promoting rhizobacteria (PGPR) that feed on root exudates can improve plant growth by various mechanisms [5–7]. A key trait of several species of PGPR is the ability to control ethylene formation using the ACC (1-aminocyclopropane-1-carboxylate) deaminase enzyme; thus PGPR act as an ACC sink. These PGPR hydrolyse the ACC exuded from the roots in the rhizosphere into ammonia and α-ketobutyrate, and stimulate the extrusion of ACC from the roots to the soil [8–10]. The lowering of ACC concentration in root tissues reduces the formation of endogenous ethylene, thus promoting plant growth. Plant resistance to drought has been reported to be enhanced by reducing ethylene-mediated inhibitory effects on plant growth [7, 11].

Ethylene affects a large number of processes involved in plant growth and development, and has a key role in plant response to biotic and abiotic stresses [12]. Ethylene synthesis occurs in three successive enzymatic conversions. Methionine is first converted to SAM (S-adenosyl methionine) followed by the formation of ACC. Increased production of ACC in roots results in higher ethylene biosynthesis by the ACC oxidase enzyme in different plant tissues [7, 10, 13, 14]. This is a typical response to the stimulation of root ethylene biosynthesis by drought. Increased ethylene formation has detrimental effects on root development and more generally on plant growth [7, 15, 16].

Ethylene affects photosynthesis and stomatal conductance, depending on ethylene production and stomatal sensitivity to this hormone [17]. In addition, ethylene interacts with other hormones, such as auxin and abscisic acid, in regulating plant response to water shortage. Drought induces a reduction in stomatal conductance (*g*_*s*_), and low *g*_s_ reduces the intercellular CO_2_ concentration (*C*_i_) and lowers photosynthesis [18–20]. During prolonged drought, plants increase the release of part of the assimilated carbon in the form of biogenic volatile organic compounds (BVOCs), such as isoprene. Isoprenoids synthesis depends on photosynthesis in well-watered conditions, however, drought is known to uncouple these two processes [21], as isoprene formation is less affected than photosynthesis in response to water deficit [18, 22, 23].

The use of PGPR as strategy to increase plant performance was selected as it is an inexpensive and easy technique that is well suited to use in developing countries. Velvet bean (*Mucuna pruriens* (L.) DC.) is an isoprene emitting leguminous crop [24] with high nutritional (overall proteins) and medicinal importance that is widely cultivated in tropical Africa and Asia [25, 26]. This bean crop is known to be well adapted to arid and semi-arid regions; therefore, the plant was inoculated with PGPR to assess its effect on growth and biomass under water deficit condition. In present study, selected ACC active PGPR strains were tested to promote growth of velvet bean in response to water deficit. Drought was quantified using the fraction of transpirable soil water technique, which allows the precise knowledge of soil water available to sustain plant transpiration [18, 21]. To our knowledge, there are no published studies describing the interactive effects between drought and PGPR inoculation on ethylene and isoprene emission.

The aim of this work was to evaluate the effect of two different strains of ACC deaminase-containing rhizobacteria on velvet bean plants under drought stress conditions. Photosynthetic performance and biomass were evaluated to verify the growth promoting effect on plant of the two selected rhizobacterial strains. Furthermore, ACC and ethylene content of plants leaves and roots were evaluated as they are key molecules involved in the mechanisms of action of ACC deaminase active PGPR. Finally, isoprene emission was measured to evaluate the plant investment in the biosynthesis of this antioxidant secondary metabolite.

## Materials and methods

### Rhizobacteria growth conditions and identification

Two previously isolated strains (HS9 and G9) of rhizobacteria were characterized for their ACC deaminase activity and IAA production through morphological and chemical assays as reported in Saleem et al. 2015, 2016 [27–28]. These PGPR were isolated from the rhizosphere soil of velvet bean (*Mucuna pruriens*) using a modified minimal medium DF salts [29] containing ACC as the sole source of nitrogen. The modified medium (pH 7.2) contained (g L^-1^) glucose (1.5), KH_2_PO_4_ (0.5), MgSO_4_^.^7H_2_O (0.1) and Fe_2_SO_4_^.^7H_2_O (0.2) and ACC (0.05). All chemicals were purchased from Sigma-Aldrich. The medium was inoculated separately with the two rhizobacterial strains. The flasks were then incubated at 29 ± 1°C for 48 h in a shaking incubator at 80 rpm. The optical density (OD) of each culture was measured spectrophotometrically at 590 nm and diluted to a value of OD = 0.7 ± 0.02 with sterilized uninoculated medium. These cultures were used for 16S rRNA gene analysis and for *in vivo* experiments on velvet bean.

To identify rhizobacterial strains, 16S rRNA gene analysis was performed. For this purpose, bacterial DNA was first extracted with an EZNA centrifuge kit (Omega Biotech Inc.). Amplification of 16S rRNA gene was performed using the method described by Dineen et al. 2008 [30]. Overall, the reaction was performed in a total volume of 20 μL using 10 pmol of each primer [27F, 5’-GAGAGTTTGATCCTGGCTCAG, and 1495R, 5’ CTACGGCTACCTTGTTACGA] (Invitrogen, Life Technologies). Polymerase chain reaction (PCR) was carried out at 95°C for 1.5 min, 35 cycles consisting of 95°C for 30 s, varying annealing temperatures for 30 s, and then 72°C for 4 min for extension phase. The annealing temperature was 60°C for the first 5 cycles, 55°C for the next 5 cycles, and 50°C for the last 25 cycles. Finally, the mixture was incubated at 72°C for 10 min and then at 60°C for 10 min.

Direct sequencing of amplicons was performed using an ABI3730 DNA analyser (Applied Biosystems, Foster City, CA, USA) using the Big Dye Terminator Kit (Applied Biosystems, Foster City, CA, USA). The 16S rRNA sequences were matched with nucleotide sequences available on GenBank database using the BLAST program [31] and MUSCLE. Multiple sequence alignment was used to align the 16S rRNA sequences obtained with the most similar orthologous sequences retrieved from the Ribosomal Database Project [32–33] (RDP; http://rdp.cme.msu.edu/). Database alignments were trimmed to eliminate poorly aligned regions and used to build Neighbour-joining dendrograms, obtained after calculation of a Kimura two-parameter distance matrix with the software Mega 5 [34]. Strains are identified at the species level when sequences share more than 97% identity with reference strains.

### Plant growth conditions and water deficit experiment

Velvet bean seeds were externally sterilized by dipping in 95% ethanol and immersing in 0.2% HgCl_2_ solution for 3 min. The seeds were washed with sterile distilled water [5] and planted in pots filled with autoclaved fluvial sand. The soil of 28 independent plants was inoculated with 15cc of each bacterial inoculum (14 plants per strain, called ‘inoculated plants’ hereafter), while 7 pots were treated with 15cc of sterilized uninoculated medium. Seven days after seed germination, seedlings were transplanted in 8 dm^3^ pots containing the same substrate. Plants were grown in a climate-controlled phytotron with an ambient day/night temperature of 25–28°C/18-20°C, a daily photoperiod of 14h, a photosynthetic photon flux density (PPFD) 1000 μmol m^-2^ s^-1^, and daily relative humidity ranging between 50–60%.

Plants were kept in well-watered conditions and fertilized with half-strength Hoagland solution each week to obtain nutrients at free access rate [35] for 30 days. The plants were on average 70 cm in height at the beginning of the water deficit treatment. All plants were watered to pot water capacity on the afternoon prior to the onset of the drought cycle, and pots were then allowed to drain the excess water overnight. The following morning, plants were weighed with a digital balance (model QS32A, Sartorius Instrumentation, Göttingen, Germany), with 1-g precision, to measure the initial weight (Initial_pot weight_) at pot water capacity. Pots were then wrapped in plastic film, to prevent soil evaporation, and weighed daily (Daily_pot weight_) during the experiment. Water deficit, expressed as a function of fraction of transpirable soil water (FTSW) [22, 36], was applied by withholding water on five plants per PGPR and water treatments. The FTSW was calculated as follows:

$$FTSW=(Daily_{pot\;weight}‑Final_{pot\;weight})/(Initial_{pot\;weight}‑Final_{pot\;weight})$$

where, Final_pot weight_ refers to the weight of water-stressed plants when stomatal conductance approached zero (i.e., end of the water deficit cycle).

### Gas exchange and isoprene emission measurements

Photosynthetic rate (*A*) and stomatal conductance (*g*_s_) were measured daily (between 10:00 am and 2:00 pm) on 5 new fully expanded leaves (one per plant) per treatment by a portable infrared gas analyzer (IRGA, Li-cor 6400XT, Lincoln, NE, USA) by enclosing a portion of a single leaf in a 6 cm^2^ gas-exchange cuvette. Measurements were performed at 400 μmol mol^-1^ CO_2_, 1000 μmol m^-2^ s^-1^ photosynthetic photon flux density (PPFD), a leaf temperature of 30°C and a relative humidity ranging between 45% and 55%. Measurements were recorded when *A* reached a steady state.

Following gas exchange measurements, the same leaves were used to determine isoprene emission by connecting the match tube outflow on the IRGA cuvette into a biphasic adsorbent trap containing 30 mg of Tenax and 20 mg of Carboxen (GERSTEL GmbH & Co.KG, Germany). Measurements of isoprene emission were performed three times: at the beginning of the stress in well-watered plants, i.e. 100% of FTSW (FTSW_100_), in the middle of the drought stress experiment (FTSW_60_) and in severely water-stressed plants, i.e. 10% of FTSW (FTSW_10_) inoculated with the two bacterial strains and uninoculated. A pump (Elite 5, A.P. Buck, Orlando, Florida, USA) was used to pass 2 L of air through each trap at a rate of 200 ml min^-1^. Measurements were made in well-watered (FTSW_100_) plants and under moderate (FTSW_60_) and severe drought stress (FTSW_10_) conditions. Traps were stored at 4°C, and then isoprene was quantified using a GC-MS (Agilent Technologies, Wilmington, DE, USA) attached to a Gerstel sampler, a thermal desorber UNIT (Gerstel Inc., Mulheim, Germany) and a HP-INNOWAX capillary column (50 m in length, 200 m i.d. and 0.4 m film thickness). The column oven temperature was kept at 40°C for the first 1 min, then increased by 2°C min^-1^ to 60°C, 3°C min-^1^ to 150°C, 10°C min^-1^ to 200°C, 20°C min^-1^ to 260°C and maintained at 260°C for 6 min. Helium was used as a carrier gas. The concentration of isoprene was calculated by comparison with the peak area of a gaseous standard. The GC-MS was calibrated using cylinders with isoprene standards at a concentration of 1 mg L^-1^ (Rivoira, Milan, Italy). The compound identification was made using the NIST library provided with the GC/MS ChemStation software (Agilent). The GC peak retention time was substantiated by analysis of parent ions and main fragments on the spectra.

### Measurement of ethylene and ACC concentration

Ethylene and ACC content were measured at the end of the experiment in both leaf and root samples of severely water-stressed plants (FTSW_10_) in soil inoculated and uninoculated plants. Five fresh leaves and five root samples were weighed immediately after being detached from the plants and sealed in glass vials with water-saturated filter paper. Vials were incubated under illumination providing a photosynthetic photon flux density (PPFD) of ~1000 μmol m^-2^ s^-1^ at 23 ± 1°C. Ethylene accumulation was detected after 60 min of incubation. The sampled leaves and roots were then oven dried (95 ± 2°C for 24 h) and weighed again for dry weight (DW) and the ethylene measurement was expressed as nl g^-1^ DW h^-1^ [37].

Ethylene content in the vials was determined using an ultra-sensitive photoacoustic laser spectrophotometer (ETD-300, Sensor Sense B.V., Nijmegen, The Netherlands) in combination with a valve control box (type VC-6, Sensor Sense B.V. Nijmegen, The Netherlands). The detector is able to detect a range from 0–5 ppmV (part per million by volume) of ethylene within a 5-s time scale [38]. In this experiment, the measurement time was five seconds with a flow of 0.25–5 dm^3^ per hour (a flow of 3 L h^-1^).

The quantity of ACC was measured after an in vitro chemical conversion of ACC into ethylene [37]. Leaf and root ACC concentration was chemically converted into ethylene and measured by the ethylene detector. Briefly, 2 g of freeze-dried leaf tissue was powdered with liquid nitrogen and extracted with 4 ml of sulfo-salicylic acid solution (5% w/v) for 30 min at 30°C. The mixture was centrifuged for 10 min at 3,090×g in a centrifuge; 1.5 ml of the supernatant was collected and to it 0.4 ml of HgCl2 solution (10 mM) and 0.2 ml of a NaOCl—NaOH (2:1, v/v) solution was added. The solution was vortexed for 5 seconds and left on ice for 5 min. Then ethylene produced inside the vial was analyzed using the ethylene detector ETD-300 described above, operating at gas flow of 3 L h^-1^. The quantification was performed using a six-point calibration curve constructed from an ACC standard (Sigma Aldrich, Milan, Italy).

### Plants biometric measurements

At the end of the drought stress experiment, the total length of the stem from the collar and the maximum length of the root of 10 plants per treatment were measured. The roots were gently washed and the surrounding soil removed. Then the roots and the above ground portion of each plant were separately oven-dried (95 ± 2°C for 24h) and weighed to record dry biomass. The number of leaves of control and inoculated plants were also counted.

### Statistical analysis

Five replicates for each treatment were measured during the experiment. Significant differences (5% level) between treatments were tested, at each sampling date, by a one-way ANOVA (post-hoc Tukey test) using SPSS software (IBM SPSS 20.0 software, SPSS Inc., Chicago, USA).

## Results

### Rhizobacteria identification

**Fig 1:**
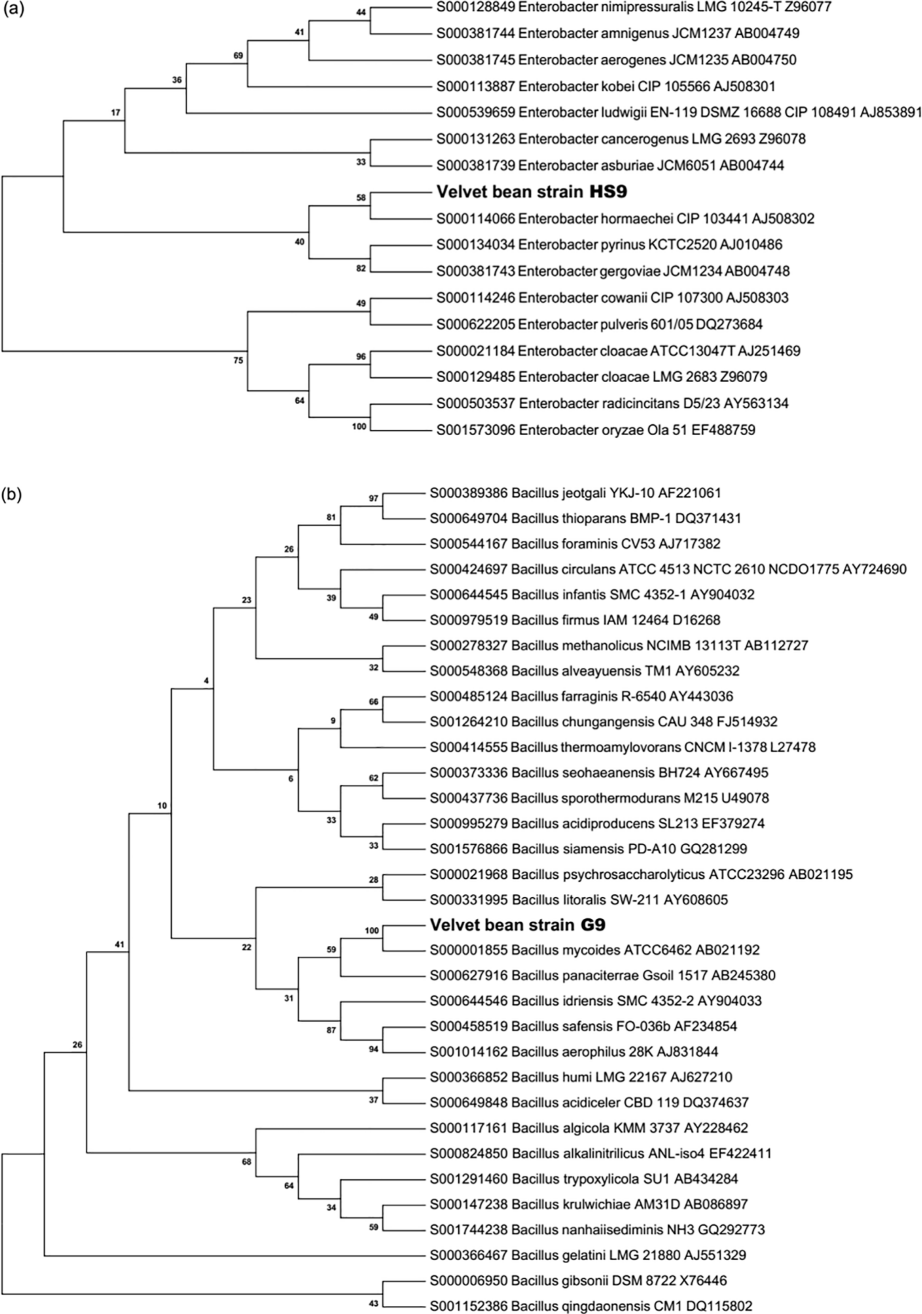
Phylogenetic dendrograms showing the relationship between the selected bacterial strains with closely related taxa of *Enterobacter* (a) and *Bacillus* (b), respectively, inferred from 16S rRNA sequences. The dendrograms were generated using the Neighbour-Joining method.

To determine the identity of the HS9 and G9 selected strains of rhizobacteria possessing ACC deaminase activity and producing IAA [28], the 16S rRNA gene region was sequenced. Strain HS9 and G9 clustered in the phylogenetic dendrogram, generated using the Neighbour-Joining method, within *Enterobacter* and *Bacillus* genera, respectively (Fig 1). However, the identification of species was not possible since the ID percentage was lower than 97% for both strains. Sequences of *Enterobacter* HS9 and *Bacillus* G9 were deposited to GenBank with the accession numbers MF348312 and MF348311, respectively.

### Photosynthesis performance

**Fig 2:**
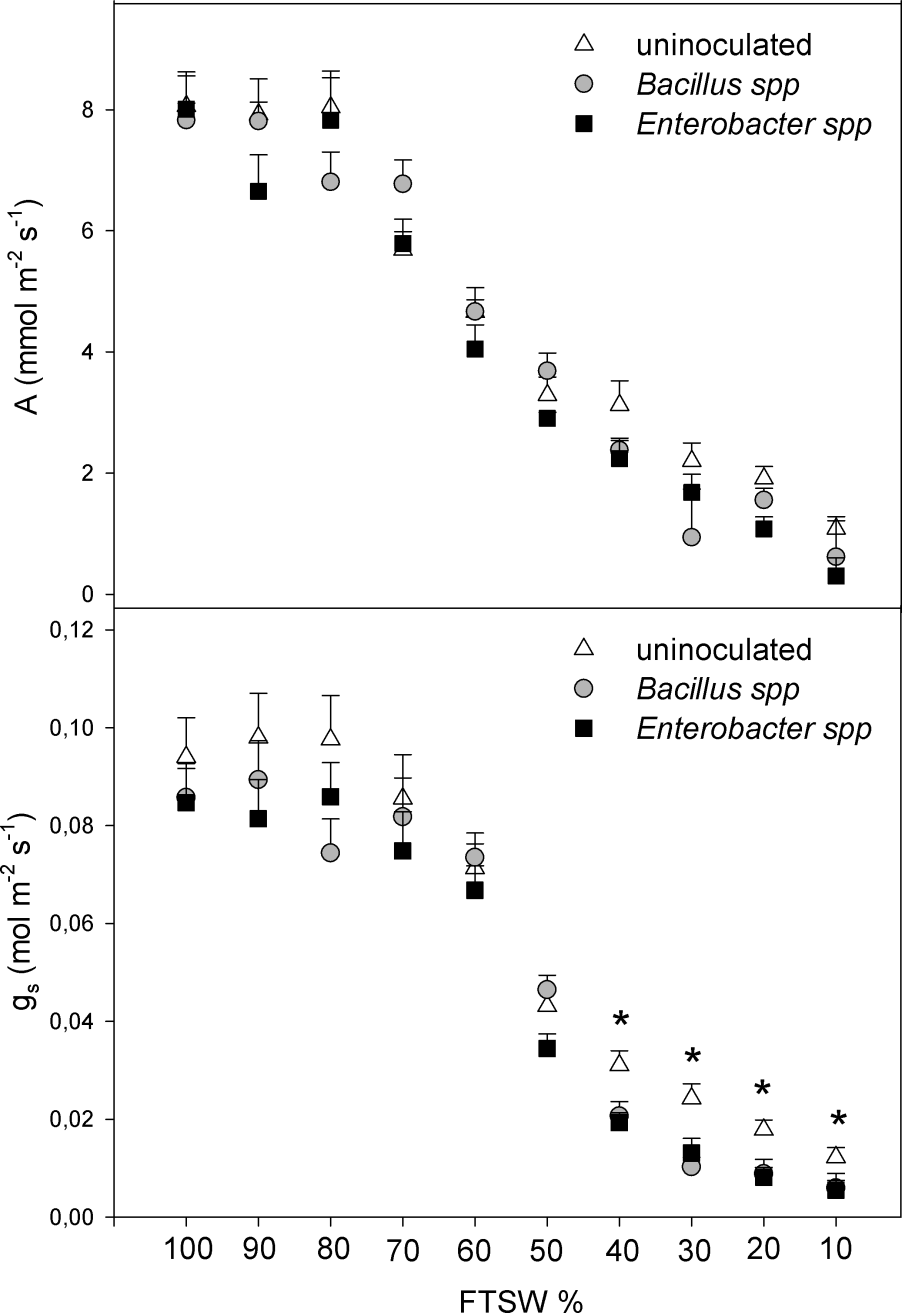
Effect of *Enterobacter* spp. and *Bacillus* spp. soil inoculation on photosynthesis (*A*) and stomatal conductance (*g*_s_) in velvet bean plants under drought stress as a function of fraction of transpirable soil water (FTSW). Values are averages ± SE of five replicates (plants). Asterisks (_*_) indicate significant differences among treatments (*P* < 0.05).

Photosynthesis (*A*) and stomatal conductance (*g*_s_) (Fig 2) of *M*. *pruriens* plants decreased as water deficit progressed. The drying cycle ended at FTSW_10_ (severe drought stress), when stomata were virtually completely closed. Similar responses in both uninoculated and inoculated plants (irrespective of the bacterial strains) were showed from *A* and *g*_*s*_. After FTSW_60_ these two gas exchange parameters began to decline rapidly. Furthermore, starting from FTSW_40_, *g*_*s*_ of non-inoculated plants showed significant higher values than inoculated plants. At FTSW_10_, *A* and *g*_s_ were on average 97–98% lower than the FTSW_100_ values in both inoculated and uninoculated plants, and no differences were observed among bacterial strains. Overall, PGPR inoculation did not significantly affect the kinetics of *A* and *g*_s_ in response to FTSW. The physiological pattern of *A* and *g*_*s*_ resulted in generally similar *C*_i_ values in both inoculated and non-inoculated plants in response to water deficit (S1 Fig) until FTSW_10_.

### Plants biometric measurements

**Fig 3:**
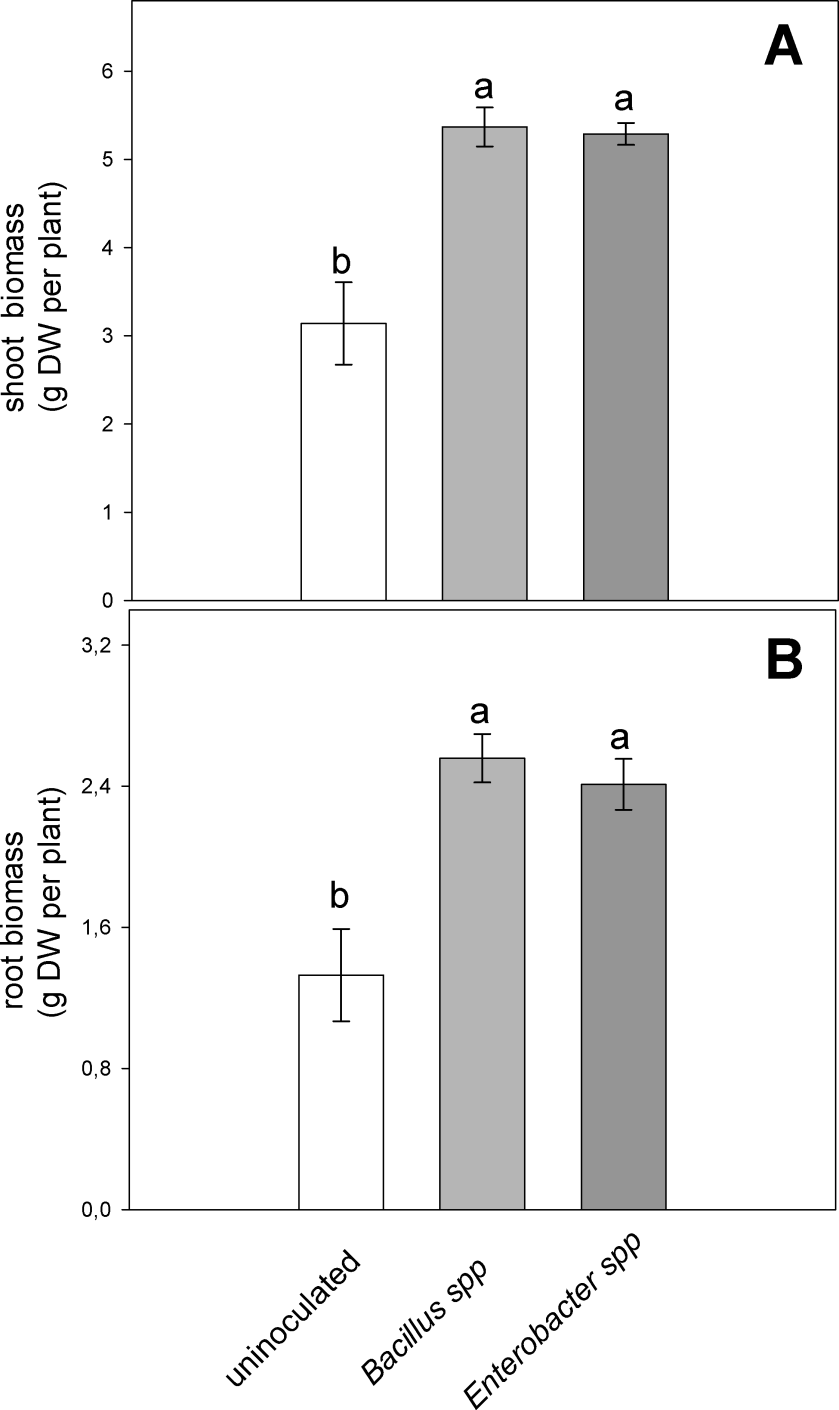
Effect of severe water stress (FSTW_10_) on shoot (A) and root (B) biomass of velvet bean plants grown in soil inoculated with *Enterobacter* spp. and *Bacillus* spp., or uninoculated. Values are mean of five plants ± SE, letters indicate significant differences among treatments (*P* < 0.05).

PGPR inoculation, with *Enterobacter* spp. and *Bacillus* spp. strains, at the end of the drought stress cycle (FSTW_10_), resulted in significant increases of root and shoot length compared to control plants (Table 1). This is also confirmed by shoot and root biomass (dry weight) showed a significant increase in comparison to non-inoculated plants (Fig 3). The number of leaves in inoculated plants were significantly higher than uninoculated plants (Fig 3). In particular, inoculated plants showed 25% and 27% longer maximum root length in G9 and HS9 respectively, than uninoculated plants. On the other hand, plant shoot total length was 28% and 18% higher in G9 and HS9 respectively than in inoculated plants. Whereas, shoot and root dry mass were increased on average by about 70% and 78%, respectively, by PGPR inoculation. No significant differences were observed for any morphological parameters between the two PGPR strains.

**Table 1:** Plant biometric measurements at the end of the experiment (day 10) of velvet bean plants grown in soil inoculated with *Enterobacter* spp. and *Bacillus* spp., or uninoculated. Values are the mean of 10 plants. Different letters indicate significant differences among treatments (one-way ANOVA, Tukey test) for each parameter (*P* < 0.05).

Bacterial strain	Total shoot length (cm)	Max root length (cm)	number of leaves
Uninoculated (control)	159.2 ^a^	39.4 ^a^	31.2 ^a^
*Enterobacter* spp. (HS9)	189 ^b^	50.2 ^b^	43.8 ^b^
*Bacillus* spp. (G9)	203.6 ^b^	49.2 ^b^	44.2 ^b^

### Measurement of ethylene and ACC concentration

**Fig 4:**
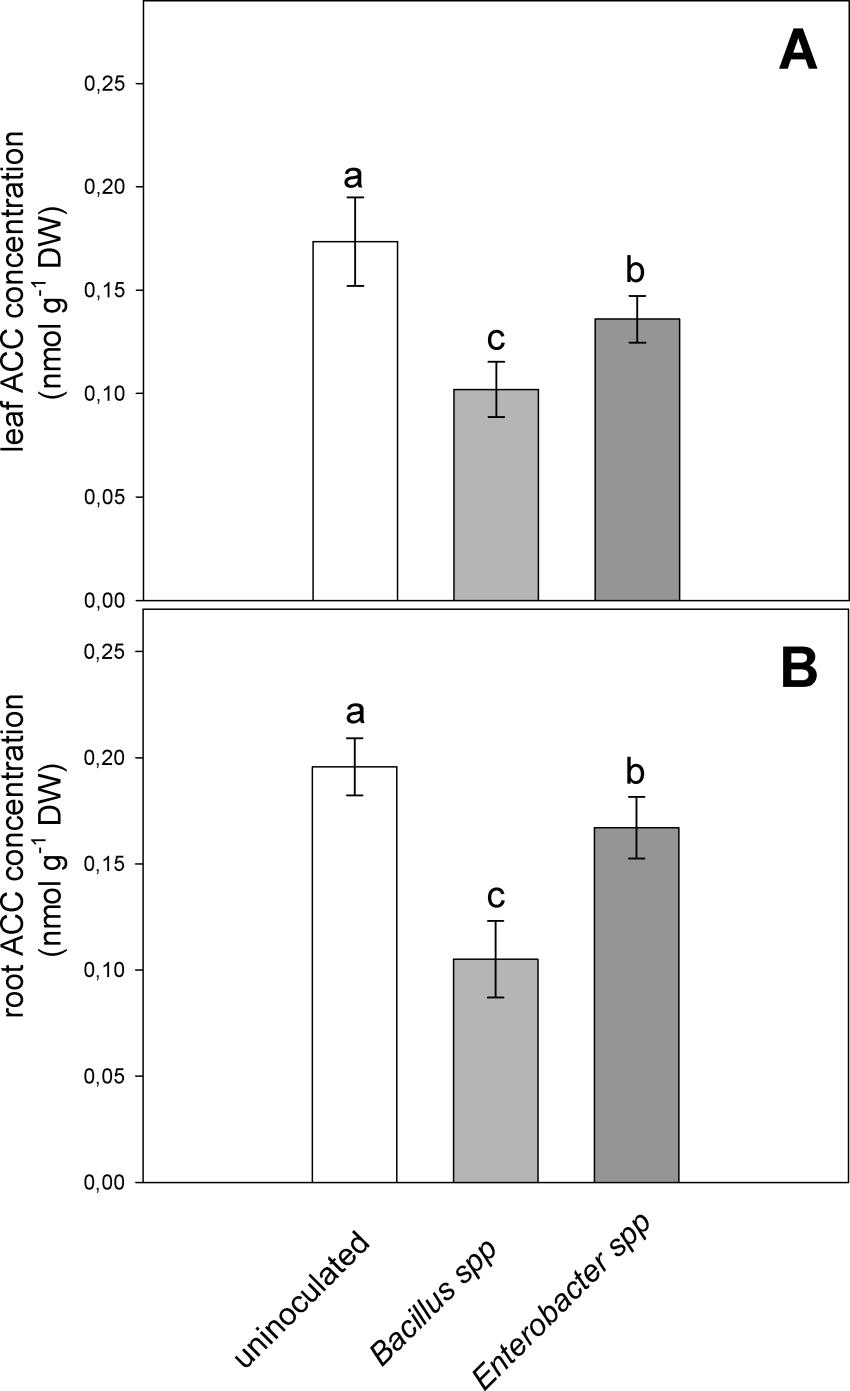
Effect of severe water stress (FSTW_10_) on ACC (1-Aminocyclopropane-1-carboxylate) concentration in leaves (A) and roots (B) of velvet bean plants grown in soil inoculated with *Enterobacter* spp. and *Bacillus* spp., or uninoculated. Values are means of five plants ± SE, letters indicate significant differences among treatments (*P* < 0.05).

Analysis of plant tissues showed that PGPR inoculation affected negatively ACC concentration in leaves and roots (Fig 4). The ACC content in leaves of plants inoculated with *Bacillus* spp. strain was reduced by 41%, while in plants inoculated with *Enterobacter* spp. strain the reduction was of 21% in comparison to the uninoculated plants (Fig 4A). A similar trend was also observed for ACC concentration in roots (Fig 4B), where decreases of about 46% and 15% of ACC concentration were observed in *Bacillus* spp. and *Enterobacter* spp., respectively.

**Fig 5:**
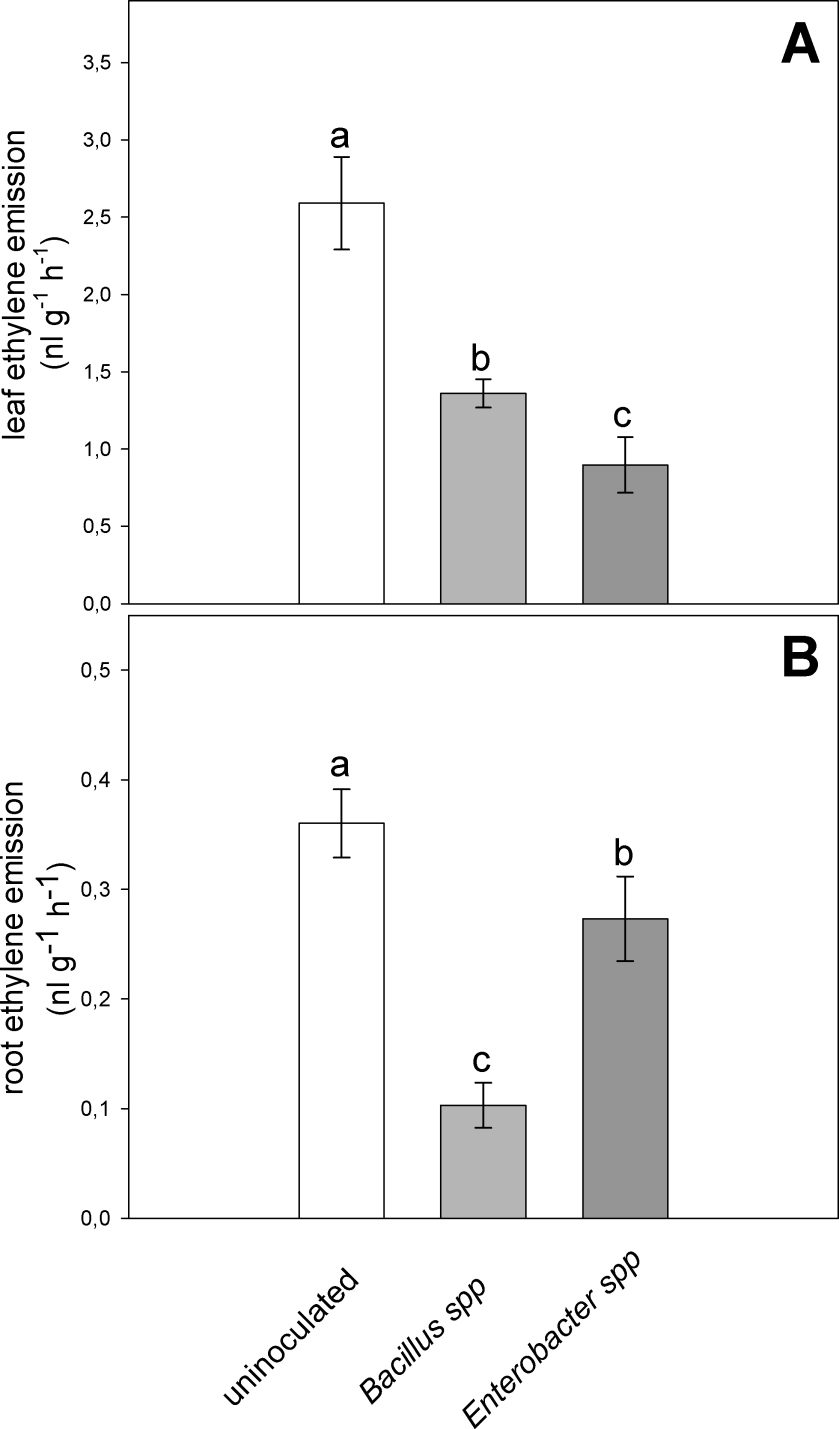
Effect of the severe water stress (FSTW_10_) on ethylene emission (nl g^-1^ h^-1^ dry weight) from leaves (a) and roots (b) of velvet bean plants grown in soil inoculated with *Enterobacter* spp. and *Bacillus* spp., or uninoculated. Values are means of five plants ± SE, letters indicate significant differences among treatments (*P* < 0.05).

Inoculation with ACC deaminase active rhizobacteria significantly reduced ethylene emission from both leaves and roots under severe water-stressed conditions (Fig 5). Leaf ethylene emission was decreased by more than 45% in plants inoculated with the *Bacillus* spp. strain, whereas the reduction exceeded 65% in plants inoculated with the *Enterobacter* spp. strain (Fig 5A) when compared to the uninoculated plants. Ethylene released by roots was also significantly reduced in response to inoculation with *Bacillus* spp. and *Enterobacter* spp. strains by 72% and 25%, respectively, compared to uninoculated plants (Fig 5B). Significant differences in ethylene emission were also recorded between the two bacterial strains, as *Enterobacter* induced a lower ethylene emission from the leaves, while the lowest root emission of ethylene was induced by the *Bacillus* bacterial strain.

### Measurement of isoprene emission

**Fig 6:**
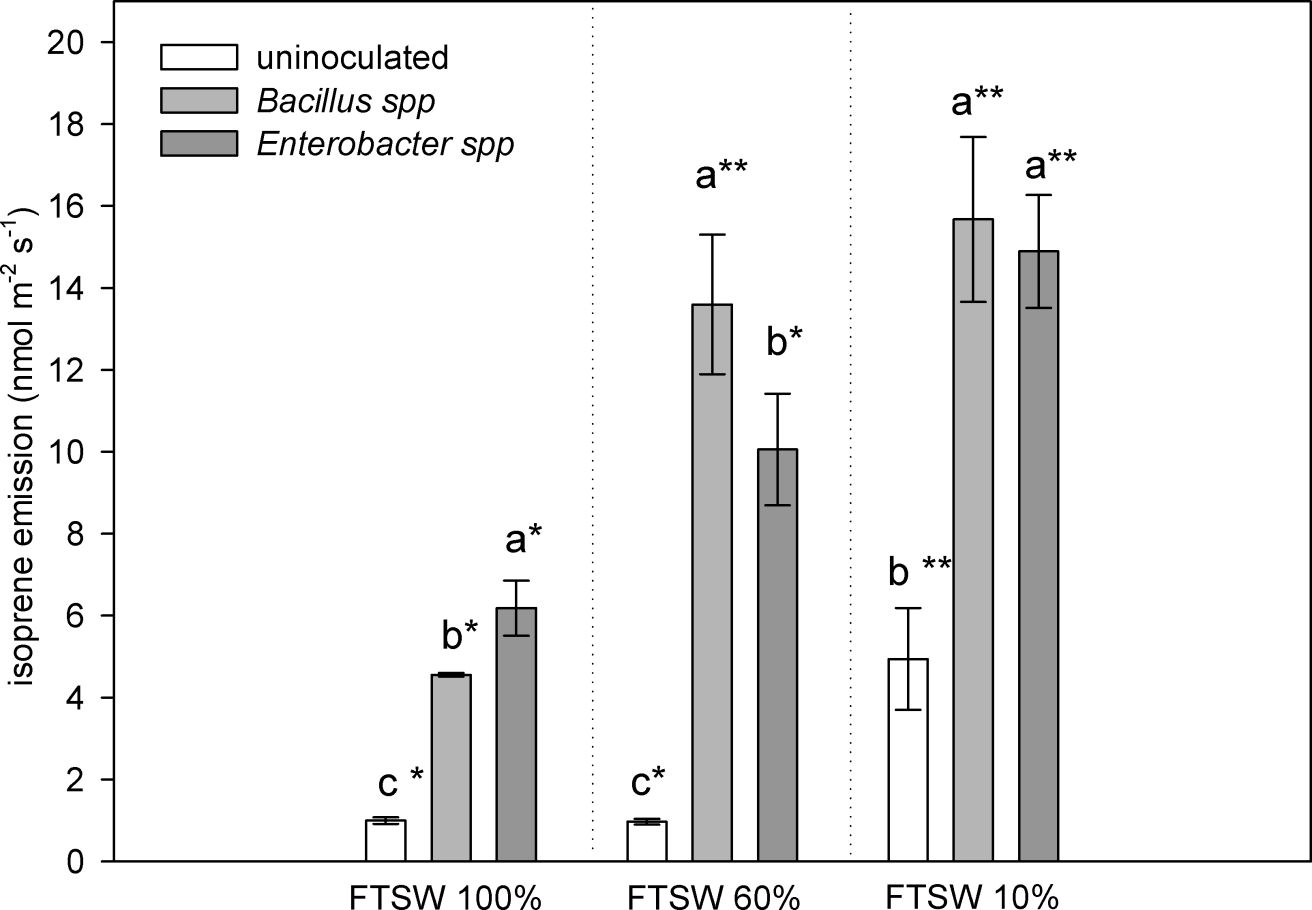
Isoprene emission from leaves of velvet bean plants grown in soil inoculated with *Enterobacter* spp. and *Bacillus* spp., or uninoculated. The measurements were made at 100%, 60% and 10% of fraction of transpirable soil water (FTSW). Values are means of five plants ± SE, letters indicate significant differences among treatments at the same FTSW, while asterisks (_*_) indicate significant difference among uninoculated, inoculated with *Enterobacter spp*. and inoculated with *Bacillus spp*. plants in the three FTSW levels (*P* < 0.05).

Isoprene emission was measured in well-watered conditions (FTSE_100_), in plants subjected to moderate (FTSW_60_) and severe (FTSW_10_) drought stress (Fig 6). Isoprene emission was higher in inoculated plants than in uninoculated plants in both well-watered and water shortage conditions. As drought stress progressed, isoprene emission increased in inoculated (from FTSW_60_) plants, and later in uninoculated plants (FTSW_10_). The isoprene emission at FTSW_10_, i.e. at the end of the drought cycle, was about five- and three-fold higher than that in well-watered plants (FTSE_100_) in uninoculated and inoculated plants respectively. At FTSW_100_ and FTSW_60_ a different effect of the bacterial strains in leaf isoprene emission was evident. In fact, in well-watered conditions (FTSW_100_) plants inoculated with the *Enterobacter* strain showed higher isoprene emission than plants inoculated with *Bacillus* bacterial strain. In contrast, at FTSW_60_ isoprene emission was higher in plants inoculated with the *Bacillus* strain than those inoculated with the *Enterobacter* strain. Both inoculated sets of plants showed significantly higher emission of isoprene than uninoculated plants. At FTSW_10_ no differences between the two strains were found (Fig 6). With respect to the well-watered initial condition, the isoprene emission of plants inoculated with the *Bacillus* strain increased significantly at FTSW_60_, while plants inoculated with *Enterobacter* strain showed the higher increment later when the drought stress was severe (FTSW_10_).

## Discussion

Several rhizobacteria have the intrinsic ability to improve the growth and drought resistance in plants [39–41]. PGPR carrying the ACC deaminase gene may be beneficial to plants under stress by reducing levels of the stress hormone ethylene within plant tissues [7, 40]. The present study demonstrated the potential to improve the growth and biomass of velvet bean under water deficit conditions of PGPR with ACC deaminase activity isolated from a rainfed farming system in Pakistan. Several researchers have reported that a variety of bacteria isolated from different environments contain ACC deaminase enzyme, and these bacteria have the ability to enhance the growth of various crop plants [7, 10, 41–45]. The bacterial genus *Bacillus* is recognized as the most abundant in the root zone of drought-adapted plants [45], since these bacteria stay as spores for their survival in water scarcity conditions [42]. However, the soils of rainfed agricultural systems, to the best of our knowledge, have not been thoroughly investigated for native beneficial bacteria to alleviate stress induced by drought in agriculture.

Photosynthesis (*A*) is generally directly coupled with stomatal conductance (*g*_s_) [4, 20]. In our study as soil dries, CO_2_ diffusion through the stomata is progressively decreased (i.e., decreasing *g*_s_) resulting in reduced photosynthetic rates as reported in previous studies [19, 20, 46–48]. ACC deaminase active rhizobacteria inoculation did not affect significantly the kinetics of *A* and *g*_s_ per unit leaf area in response to FTSW. These results are in keeping with previous reports [49, 50] showing that rhizobacterial inoculation did not significantly influence *A* under stress. The present study in which ACCd *Bacillus* and *Enterobacter* strains promoted the root and shoot growth of velvet bean under drought stress (Fig 3) concurred with earlier reports in which improved root growth was observed upon inoculation with ACCd active rhizobacteria [3, 51]. The improved root growth might facilitate increased water-uptake from a larger volume of soil. In response to soil drying, plants release chemicals signals, (e.g., abscisic acid, ACC, ethylene) [52–54] which act as cascade of stress signals from root to shoot through the transpiration stream [3, 14]. Ethylene plays different but interchangeable roles in regulating *g*_s_ [3]. Earlier studies reported ethylene induced stomatal closure under drought conditions [3, 52, 54], while inoculation with *Variovorax paradoxus* 5 C-2 (a gram negative, beta proteobacterium) restored the stomatal sensitivity to soil drying [14]. Stomatal sensitivity was enhanced under severe drought, likely as a result of the reduced antagonistic effect of ethylene on ABA [55]. Thus, ACCd active rhizobacteria can reduce the inhibitory effects of drought, through the cascading stress signals from root to shoot [56]. Results of our study provided evidence that inoculation with PGPR strain producing ACCd enzyme significantly reduce the ethylene emission from roots and leaves of velvet bean plants. The ethylene emission from inoculated plants was significantly lower when compared to non-inoculated plants, which can be ascribed to lower ACC content in plant tissues, especially in the roots. In fact, it has already been observed that stress-induced ethylene acts as an inhibitor of plant growth, particularly primary root elongation and lateral root formation at early stages of plant development [57–59]. Moreover, the PGPRs with ACCd activity can enhance root growth by lowering the stress hormone ethylene [7, 57].

As ACC is the precursor of ethylene, we hypothesize that the specific PGPR hydrolyzes the plant exuded ACC in the root-zone to maintain its equilibrium between root, rhizosphere and bacterial cell. The PGPR convert ACC into α-ketobeutyrate that results in decreased ACC and ethylene concentration in plants [7, 56, 60, 61]. The results of the present study present further evidence to link plant growth and microbial ACC deaminase activity. Under severe drought stress (FTSW_10_), a significantly lower ACC concentration was observed inside the leaves and roots of inoculated plants when compared with uninoculated plants. These findings indicate that PGPR containing ACC deaminase enzyme can reduce ACC content inside plant tissue, and therefore minimize its associated negative impacts on plant growth and development by maintaining the ACC balance in the plant and rhizosphere [43, 62]. Usually less ACC is exuded under transpiring conditions compared with non-transpiring conditions [43]. Soil drying increases the concentration of ACC inside the xylem and root [14, 56]. This mechanism of action supports the results of the present study as a greater concentration of ACC was found inside the root and leaf of the uninoculated stressed plants at FTSW_10_.

The reduction in ACC concentration acts as long distance chemical signal and limits the leaf ethylene emission [56, 61]. However, it is not yet clear whether the ACC deaminase activity of PGPR may have a systemic effect on ethylene emission. This issue has been addressed in our study by measuring the ACC concentration and ethylene evolution in both leaves and above-ground parts of the velvet bean plants. In our study, leaf ethylene was reduced (> 50%) in response to inoculation, indicating that PGPR have systemic effect. Although rhizobacteria do not promptly enhance the water and nutrient provision to plants, continuous interaction supports plant growth, particularly by enhancing root development [7, 39, 63]. In this study, inoculation with *Bacillus* and *Enterobacter* strains significantly improved root and shoot length and biomass of velvet bean under progressive drought. Previous finding showed that both bacterial strains, had the ability to produce IAA [28] which may also play a significant role in improving plant growth and biomass under drought condition [64]. These findings clearly illustrate that selected PGPR strains isolated from rainfed areas could be highly effective in promoting plant growth under drought conditions. *Bacillus spp* seems to perform better in regulating ACC content in both leaf and root, probably reducing ACC accumulation in the roots by acting on ACC synthase (ACS). *Enterobacter spp* seems to affect ACC accumulation in both leaf and root, albeit to a less extent compared to *Bacillus spp*. In order to compare the global balance of ethylene biosynthesis, we computed the ratio between ethylene emission and ACC concentration, which can be related to the enzymatic activity of ACO (ACC oxidase) (S2 Fig). The ratios show that ACO activity is not affected by *Bacillus spp* in both leaf and root compared to control plants, while inoculation with *Enterobacter spp* seems to decrease ACC oxidation to ethylene only in the leaves. In conclusion, *Bacillus spp* show a more clear action on plant ethylene bioisynthesis, by decreasing ACC levels more efficiently than *Enterobacter spp*. *Enterobacter spp* could have a further indirect effect reducing leaf ethylene emission, however more investigations would be necessary to elucidate the mechanism.

Many plants tend to release increasing assimilated carbon as isoprene under stress [18, 21, 22, 65], which behaves as a plant defense system depending on the intensity and stress duration [65]. Indeed, isoprene may effectively counter the photo-oxidative stress plants face during drought periods [66, 67]. In our study, isoprene emission increased in response to FTSW in plants inoculated with ACCd active rhizobacteria. This association pattern confirms once again that isoprene is less sensitive to water deficiency compared to *A* [21]. Thus, it can be speculated that the stimulation of isoprene biosynthesis in response to increasing drought may have increased the tolerance of velvet plants inoculated with ACCd active rhizobacteria to drought.

## Conclusion

The present study demonstrated that the decrease of ACC content in plant tissue and the subsequent inhibition of ethylene release in plants inoculated with rhizobacteria producing ACC-deaminase and IAA, increased the drought tolerance of velvet bean. Both PGPR strains specifically enhanced whole plant biomass and isoprene emission. Upon inoculation *Bacillus* spp. showed improved performance than *Enterobacter* spp. strain, though both performed far better than uninoculated plants. *Bacillus* spp. may be considered to be slightly more promising strains for the reduction of plant ACC and root ethylene level. The present work contributes to knowledge on the interactive role of ACC deaminase activity rhizobacteria in terms of plant physiology and carbon allocation in plants exposed to environmental stresses. Thus, the use of such PGPR could be very effective in improving plant growth and yield under climate change conditions in drought prone areas.

## Supporting information

S1 Fig The ratio between ethylene emission and ACC concentration from leaves of velvet bean plants grown in soil inoculated with *Enterobacter* spp. and *Bacillus* spp., or uninoculated. This ratio has been used to estimate the activity of ACC oxidase (ACO activity). The asterisk (_*_) indicates the significant difference in ACO activity in the leaves of plants inoculated with *Enterobacter spp* compared to uninoculated plants and plants inoculated with *Bacillus spp*. (*P* < 0.05).
(TIF)

S2 Fig Effect of Enterobacter spp. and Bacillus spp. soil inoculation on intercellular CO2 concentration (Ci) in velvet bean plants under drought stress as a function of fraction of transpirable soil water (FTSW). Values are averages ± SE of five replicates (plants). Asterisks (*) indicate significant differences among treatments (P < 0.05).
(TIF)
