# Supplementary figures and images for: Drought response of Mucuna pruriens (L.) DC. inoculated with ACC deaminase and IAA producing rhizobacteria
Source: PLoS One. 2018 Feb 15;13(2):e0191218. doi: 10.1371/journal.pone.0191218 (PMC5814102; doi:10.1371/journal.pone.0191218)

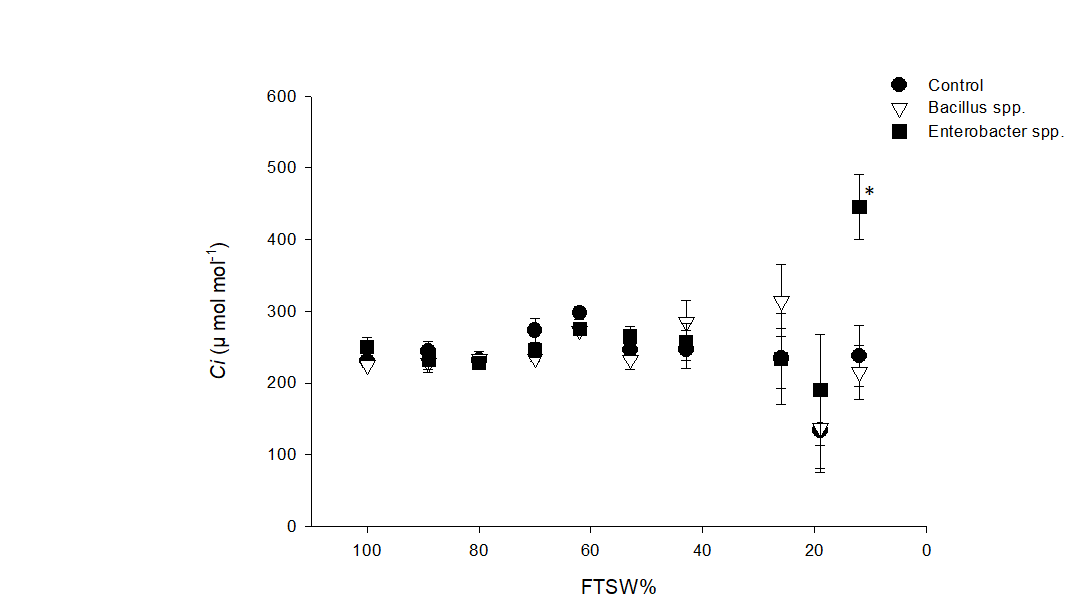

Supplement: S1 Fig — This ratio has been used to estimate the activity of ACC oxidase (ACO activity). The asterisk (*) indicates the significant difference in ACO activity in the leaves of plants inoculated with Enterobacter spp compared to uninoculated plants and plants inoculated with Bacillus spp. (P < 0.05). (TIF) [file pone.0191218.s001.tif]
